# Structural characteristics of humic substances in buried ancient paddy soils as revealed by ^13^C NMR spectroscopy

**DOI:** 10.1007/s10653-019-00297-4

**Published:** 2019-04-23

**Authors:** Pei Liu, Weijun Zhou, Haojie Cui, Jie Tan, Sheng Cao

**Affiliations:** College of Resources and Environment, Hunan Agricultural University, Changsha, 410128 China

**Keywords:** Ancient paddy soils, Solid-state ^13^C cross-polarization magic-angle-spinning nuclear magnetic resonance, Fulvic acid, Humic acid

## Abstract

The study of organic matter in ancient paddy soils is helpful for understanding the influence of human activities on soil carbon sequestration and global climate change. However, little information on the spatial distribution and structural characteristics of the humic substances (HS) in ancient paddy soils is available. The spatial distributions of humic acids (HAs) and fulvic acids (FAs) in ancient paddy soils and modern cultivated paddy soils at the Shanlonggang site on the Liyang Plain were investigated, and the associated structures were characterized by using ^13^C nuclear magnetic resonance (NMR). The ^13^C NMR spectra revealed the following carbon types in HAs and FAs in both types of paddy soil in order of decreasing abundance: O-alkyl carbon (ranging from 39.7 to 51.8% and from 42.6 to 50.9%, respectively) ≥ alkyl carbon (ranging from 16.8 to 23.5% and from 15.7 to 22.4%, respectively) ≈ carboxyl carbon (ranging from 13.3 to 19.3% and from 16.9 to 22.0%, respectively) > aromatic carbon (ranging from 12.8 to 23.5% and from 10.0 to 17.2%, respectively). Moreover, the degree of aromaticity of HA was higher than that of FA in both soil samples. The humic constituents of the buried ancient paddy soils were less aromatic and oxidized than those of the modern cultivated paddy soils. The organic carbon in the ancient paddy soils was also less aromatic and oxidized than that in the modern cultivated paddy soils, suggesting that the structures of the HS in the ancient paddy soils were relatively simple. The results of this study provide new insights into the effect of secondary paddy soil formation on the spatial distribution, structural characteristics, and stability mechanisms of the HS in ancient paddy soils.

## Introduction

Since the discovery of paddy remains and paddy farming implements in Hemudu (You 1978; Greenland 1997), the origin and spread of paddies have become the focus of research in archeology, agronomy, genetics, soil science, and environmental studies. In recent decades, the research on ancient paddy soils has mainly focused on the formation and evolution of ancient paddy soils, soil fertility characteristics (Wu et al. 2016), nutrient release, quality characteristics, biological characteristics (Zhang et al. 2016; Li et al. 2012), silicon morphology and mineralization (Cheng et al. 2011), and other aspects. The distribution of soil organic carbon (SOC) and the chemical structures and functional group compositions of humic substances (HS) in different paddy soils that are typical in China have been explored with the aid of advanced solid-state ^13^C nuclear magnetic resonance (NMR) techniques (Xu et al. 2017; Ci et al. 2009). However, little information on the spatial distribution, transformation, and structural characterization of the humic components in ancient paddy soils is available.

HS are natural organic materials that are ubiquitous in water, sediment, and soil. HS are the main component of soil organic matter (SOM) and play an important role in soil structure modification and soil fertility improvement, and functional groups of soil clay fractions play a key role in determining the adsorption affinity and hence retention of dissolved organic carbon in soils (Mandeep et al. 2017). HS commonly form organic–inorganic complexes to improve their stability, which reduces their greenhouse gas emissions, by combining with iron, aluminum oxides, and clay minerals in soils (Jung et al. 2005; Majzik and Tombacz 2007; Nimmagadda and Mcrae 2007). The HS in soils commonly consist of fulvic acids (FAs), humic acids (HAs), and humin. FAs are composed of low molecular weight compounds with simple structures and high water solubility, whereas HAs consist of high molecular weight compounds with complex structures and poor water solubility. Newly formed HS tend to be FAs, which can be precursors to HAs, and these HS decomposed two times faster than HAs during a 1-year incubation (Tate 1987; Qualls 2004). Therefore, the quality of HS could be reflected by the FA/HA ratio, and the stability of HS in soils is closely related to their distribution in soils and structural characteristics (Egli et al. 2008; Dou et al. 2008; Mikutta et al. 2009).

Solid-state ^13^C cross-polarization magic-angle-spinning (CPMAS) NMR can reflect the structural features without impairing the chemical composition of HS and is frequently used for quantitatively analyzing the structure of HS (Zhang 2000; Wang et al. 2002). NMR spectra have revealed that HS contain four types of C functional groups, alkyl (lipids, waxes, and aliphatic hydrocarbons), O-alkyl (carbohydrates), aromatic (lignin and polyphenols), and carboxyl (peptides and organic acids) groups, that can be linked to the stability of HS (Hopkins and Chudek 1997). The FA skeleton is composed of aliphatic hydrocarbons, and carboxyl groups are the main functional group. HA is composed of mostly aliphatic hydrocarbons as a fixed component and very few aromatic components (Zuo and Wen 1994; Cook and Landford 1998). The degree of aromaticity of HA and FA in soils is mostly between 30 and 45% and between 10 and 20%, respectively, and the degree of aromaticity of HA in different soils is greater than that of FA (Schnitzer et al. 1991; Dai et al. 2004; Wu et al. 2006).

Liyang Plain is one of the world’s paddy origins and communication centers (Zhang et al. 2007), where ancient paddy soil formation is affected by both ancient and modern soil-forming processes. In addition, the ancient conditions “to plow with fire and to weed with water” are significantly different from modern mechanized cultivation and the tillage application of chemical fertilizers. However, the influence of the above soil formation process on the spatial distribution and structural characterization of HS in ancient paddy soils is still not fully understood (Lee and French 2016; Wu et al. 2016). In the present work, soil samples collected from Shanlonggang archeological station on the Liyang Plain were used to study the spatial distribution of humus components in paddy soils on the Liyang Plain. Moreover, the ^13^C NMR technique was used to analyze the functional C group contents of HAs and FAs in the different soil layers of buried ancient paddy soils and modern cultivated paddy soils.

## Materials and methods

### Soil sampling

**Fig. 1:**
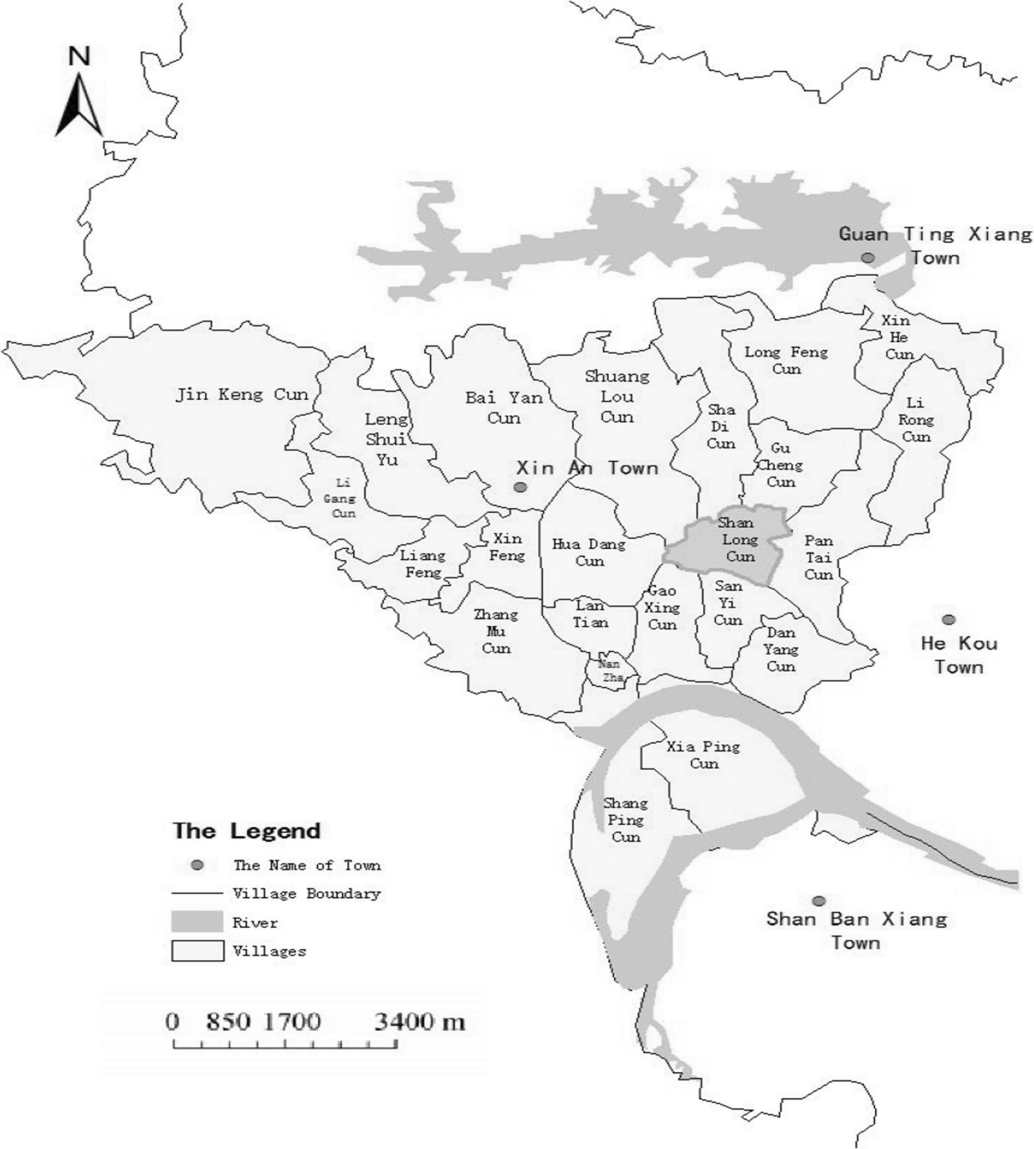
Map of the study area

**Fig. 2:**
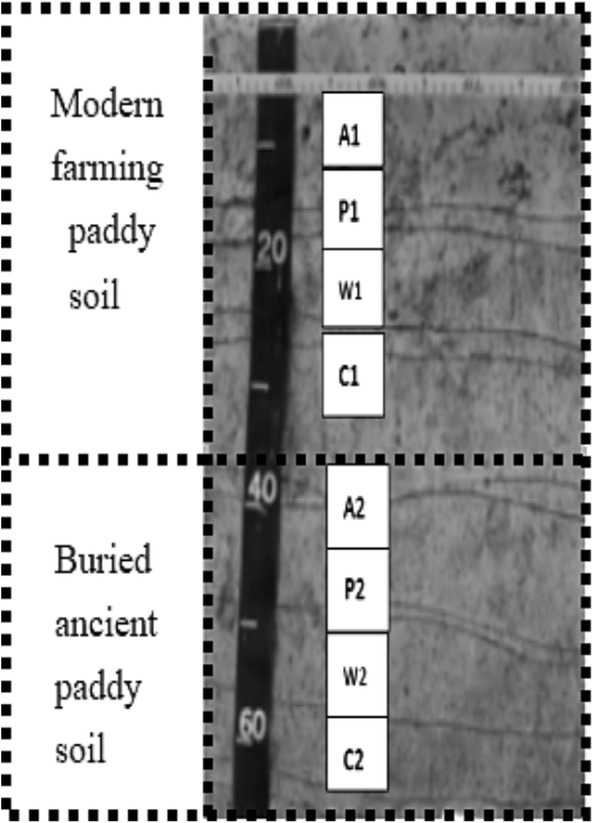
Ancient paddy soil profiles at Shanlonggang on the Liyang Plain

Soil profile samples excavated by the Hunan Provincial Institute of Archeology were collected from the archeological section (Fig. 1). According to the characteristics of the soil profile and age, the profiles were divided into two typical dating layers (Fig. 2): The layer from 0 to 39 cm was considered modern farming paddy soil, and the layer from 39 to 67 cm was considered buried ancient paddy soil (3000 years ago). Each dating layer was divided into four soil layers (A = plow layer, P = plow pan, W = waterloggogenic horizon, and C = parent material) in the modern farming paddy soils (A1–C1) and the buried ancient paddy soils (A2–C2). The soil samples were collected by layer from bottom to top and air-dried. The dried soil samples were passed through 10- and 100-mesh sieves and kept in glass bottles for later use. The basic physical and chemical properties of the soil samples are listed in Table 1.

**Table 1:** Basic properties and the humus composition of the ancient paddy soils at Shanlonggang on the Liyang Plain

Profile	Soil layer	Depth (cm)	pH	Organic carbon (g kg^−1^)	HA		FA		HA/FA
					Content (g kg^−1^)	Percentage occupied carbon (%)	Content (g kg^−1^)	Percentage occupied carbon (%)	
Modern farming paddy soil	A1	0–16	7.50	10.85	2.21	20.37	1.84	16.96	1.20
	P1	16–18	7.40	6.15	1.40	22.76	0.72	11.71	1.94
	W1	18–27	7.10	5.22	1.34	25.67	0.40	7.66	3.35
	C1	27–39	7.30	5.10	1.47	28.82	0.67	13.14	2.19
Buried ancient paddy soil	A2	39–48	7.20	7.90	1.99	25.19	1.10	13.92	1.81
	P2	48–54	7.10	7.13	2.07	29.03	1.14	15.99	1.82
	W2	54–61	7.00	7.08	2.13	30.08	0.78	11.02	2.73
	C2	61–67	6.9	6.15	2.00	32.52	0.66	10.73	3.03

*A* Plow layer, *P* plow pan, *W* waterloggogenic horizon, *C* parent material

### Extraction and purification of the humus components

Crude extraction of the soil humus components: The major humus components of the HA and FA were extracted by the Pollo modification method (Cook and Landford 1998). A 1000-g soil sample was added to 1000 mL of 0.1 mol L^−1^ NaOH solution and extracted for 24 h under N_2_ at room temperature and filtered. The residue was continuously extracted three times, and the final residue was discharged. All extraction solutions were combined and then acidified with 2.5 mol L^−1^ HCl to a pH of 1.5. The FAs were separated from the HAs by centrifugation at 3000 r min^−1^; the upper clear liquid contained FAs, and the precipitated solid contained HAs.

1. HA purification: The HAs were purified by using a small amount of 0.1 mol L^−1^ NaOH, titrated to a pH of 7 with 1 mol L^−1^ HCl, centrifuged at a speed of 3000 r min^−1^, placed in a semipermeable membrane, and dialyzed with distilled water for 5–10 days. The HAs were obtained and freeze-dried and then used for NMR characterization (Dou 2010).
2. FA purification: The FAs were purified by using active carbon for adsorption, washed with 0.2 mol L^−1^ NaOH, titrated to pH 7 with 1 mol L^−1^ HCl, placed in a semipermeable membrane, and dialyzed with distilled water for 5–10 days. The FAs were obtained, freeze-dried, and then characterized by NMR (Dou 2010).

### CPMAS ^13^C NMR measurement

The CPMAS ^13^C NMR spectra were acquired with a Bruker Avance III 400WB spectrometer (Bruker Inc., Germany) operating at a ^13^C resonating frequency of 100.18 MHz. Sixteen samples were packed in 4-mm zirconia rotors at a spinning speed of 8 kHz; the contact time was 6 ms, and the pulse delay time was 1 s. Approximately 5000 scans were collected for the samples. The recycle delay (RD) was chosen after evaluation of the T1(H) values (data not reported) so that the RD > 5T1(H). A variable contact time (VCT) pulse sequence was applied with a 1H ramp to take into account the inhomogeneity of the Hartmann–Hahn condition at high rotor spin rates. An average spin lock frequency of 60 MHz was applied during the ramped cross-polarization time. We also verified that there were no changes in the spectra acquired when an unramped CP sequence was used at the same rotor spin rate as that used for the ramped CP experiments. The contact times in the VCT sequence were 0.035, 0.045, 0.500, 0.750, 0.850, 1.0, 1.5, 2.0, 3.5, 5.0, and 6.0 ms.

### Data statistical analysis

1. Microsoft Excel 2007 (Microsoft Corporation, USA) was used for the data summary and analysis, and the ^13^C NMR spectra were obtained using MestreNova software (Mestrelab Research S.L., Spain).
2. The NMR spectra of HS could be divided into four resonance regions, namely 0–50 ppm (alkyl carbon), 50–110 ppm (O-alkyl carbon), 110–160 ppm (aromatic carbon), and 160–230 ppm (carboxylic carbon) (Li et al. 2012). The total intensity of aromaticity and aliphaticity of each sample was calculated using the following equations (Schnitzer et al. 1991):
  1 $$\text{Aliphaticity}(100\% ) = \frac{{\text{Peak}\;\text{areas}\;\text{of}\;\text{aliphatic}\;C}}{{\text{Peak}\;\text{areas}\;\text{of}\;\text{aliphatic}\;\text{and}\;\text{aromatic}\;C \times 100}}$$
  2 $$\text{Aromaticity}(100\% ) = \frac{{\text{Peak}\;\text{areas}\;\text{of}\;\text{aromatic}\;C}}{{\text{Peak}\;\text{areas}\;\text{of}\;\text{aliphatic}\;\text{and}\;\text{aromatic}\;C \times 100}}$$
3. Multivariate statistical analysis
  Two CPMAS datasets consisting of *n *× 4 matrices were developed to compare the HAs and FAs. The *n* rows in each matrix represented the eight soil layers (A1–C1, A2–C2). The four columns of the datasets were the relative spectral areas that were calculated as reported above. The four-dimensional vector for each *n *× 4 matrix was analyzed by principal component analysis (PCA) of the mean-centered data. PCA was conducted using the statistical software package Genstat (19.1) (VSNi, England).

## Results

### Spatial distribution of the soil humus components

The organic carbon content in the paddy soil profile at the Shanlonggang site varied from 5.10 to 10.85 g kg^−1^ (as shown in Table 1**)**. The organic carbon contents in the modern cultivated paddy soils and buried ancient paddy soils both decreased with increasing soil depth. For the modern cultivated paddy soil profile, the organic carbon content noticeably decreased from 10.85 (plow layer) to 5.10 g kg^−1^ (parent material). However, the buried ancient paddy soil profile presented a slight decrease from 7.90 (plow layer) to 6.15 g kg^−1^ (parent material). These results indicate that the accumulation rate of organic carbon in the modern cultivated paddy soils was faster than that of the buried ancient paddy soils, and the organic carbon content of the buried ancient paddy soils tended to become stable with increasing years of farming.

Table 1 illustrates that the HA contents ranged from 1.34 to 2.21 g kg^−1^ in the paddy soil profile at the Shanlonggang site, and the plow layer of the modern cultivated paddy soils had the greatest HA content. For the modern cultivated paddy soils, the HA contents decreased significantly with increasing soil depth, from the plow layer (2.21 g kg^−1^) to the waterloggogenic horizon (1.34 g kg^−1^), and then increased slightly with increasing soil depth to the parent material layer (1.47 g kg^−1^). However, the HA contents in the four layers of the buried ancient paddy soil increased slightly with increasing soil depth (1.99–2.13 g kg^−1^), indicating that the distribution of HA in the buried ancient paddy soils was more uniform. The ratio of HA to organic carbon in each layer of the buried ancient paddy soils varied from 25.19 to 32.52%, which was higher than that of the modern paddy soils from 20.37 to 28.82% (as shown in Table 1).

The FA contents in the paddy soil profiles at the Shanlonggang site varied from 0.40 to 1.84 g kg^−1^, and the plow layer of the modern cultivated paddy soils had the highest FA contents. For the modern cultivated paddy soils, the change in the FA contents was similar to that in the HA contents. The FA contents first decreased drastically with increasing soil depth from the plow layer (1.84 g kg^−1^) to the waterloggogenic horizon (0.4 g kg^−1^) and then increased slightly with increasing soil depth, further increasing to the parent material layer (0.67 g kg^−1^). However, the FA contents in the plow layer and plow pan layer of the buried ancient paddy soils were similar, and the contents were 1.10 and 1.14 g kg^−1^, respectively. With increasing soil depth, the FA contents slowly decreased from 1.14 g kg^−1^ in the plow pan layer to 0.66 g kg^−1^ in the parent material layer. These results suggest that the change in FA contents in the buried ancient paddy soil profile was less than that in the modern paddy soil profile, especially for the plow layer and plow pan layer. In summary, the average ratio of FA to organic carbon in the various soil layers of the buried ancient paddy soils was 12.92% (ranging from 15.99 to 10.73%), which was slightly greater than that of the modern cultivated paddy soils (average ratio 12.37%, ranging from 16.95 to 7.66%).

The ratio of HA/FA, as one of the major indicators for soil activity and the humification of SOM (McKeague et al. 1986; Gennadiyev 1998), was calculated for both paddy soil profiles, and the results are presented in Table 1. The ratio of HA/FA ranged from 1.2 to 3.35 g kg^−1^ and from 1.81 to 3.03 g kg^−1^ for the buried ancient paddy soils and the modern cultivated paddy soils at the Shanlonggang site, respectively. With the increase in the soil depth, the two groups of paddy soils showed an increasing trend in the HA/FA ratio. However, the increasing trend in the buried ancient paddy soil profile was less than that of the modern paddy soil profile, and the average HA/FA ratio of the buried paddy soils was 3.13, which was higher than that of the modern paddy soils (2.17).

### ^13^C NMR spectra of the soil HA

**Fig. 3:**
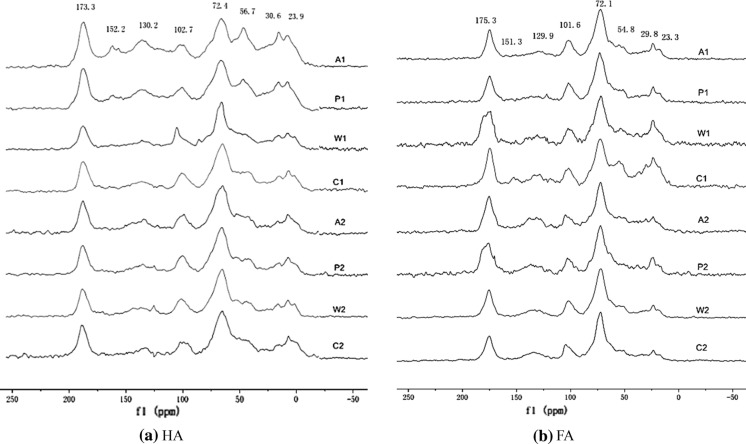
CPMAS ^13^C NMR spectra of HA and FA in paddy soil profile of the Shanlonggang site

The ^13^C NMR spectra of the HA contained in each layer of the paddy soils at the Shanlonggang site on the Liyang Plain are shown in Fig. 3. The NMR spectra revealed that the HS in each layer of the paddy soils contained four types of C functional groups: alkyl (lipids, waxes, and aliphatic hydrocarbons), O-alkyl (carbohydrates), aromatic (lignin and polyphenols), and carboxyl (peptides and organic acids) (Hopkins and Chudek 1997). The wide shoulders of the methoxyl C (50–60 ppm) and di-O-alkyl C (90–110 ppm) peaks overlapped with the O-alkyl C peak range. In the alkyl C zone, there were some common peaks near 20 and 30 ppm, which may be contributions from branched-chain and short-chain alkyl carbons (Andreia et al. 2010). In the O-alkyl region, the spectra exhibited a peak near 56.7 ppm due to methoxyl C, which is related to lignin and its analogs. The strongest of these peaks appeared near 72.4 ppm and was due to carbohydrates, and the peak near 102.7 ppm corresponds to the anomeric carbon atoms of carbohydrates, which usually produce a band between 90 and 105 ppm (Robert et al. 1998); this peak could also be due to the aliphatic carbon atoms attached to the protonated carbon atoms of phenols. The bands between 110 and 160 ppm correspond to aromatic and phenolic carbons, and the peaks in this region mainly correspond to aromatic and olefinic carbons (Wooten 1995). The intensity of the peaks varied, but generally, the weaker peaks were centered near 130 ppm, indicating ring carbons in which the ring contains a strong electron donor, such as oxygen or nitrogen. In the aromatic region, the peak at 150 ppm was weak, and the chemical shift corresponds to aromatic carbons substituted with functional groups containing oxygen and nitrogen, such as –OCH, –OH, –OR, or –NH_2_ (Poutanen and Morris 1985). The main peak near 130.2 ppm was attributed to –COOH- or –COOMe-substituted aromatic carbons, and the small signal peak near 152.2 ppm was due to phenolic hydroxyl groups. In the carboxylic region, the strong peak that appeared near 173.3 ppm corresponds to carboxylic acids, amides, and carbon ethers (Chen et al. 2007; Wu et al. 2014). The carbonyl region is between 160 and 220 ppm, and there was considerable overlap of the chemical shifts in this region due to the large number of chemical components that contain a carbonyl group; all spectra of each layer of the paddy soils showed chemical shifts of the bands between 160 and 190 ppm, and the strong peaks in this region correspond to carbonyls of carboxylic acids or carboxylate salt esters or peptides (Robert et al. 1998).

The relative concentrations of the various groups in the modern farming paddy soils and buried ancient paddy soils were calculated by integrating the areas of the corresponding peaks into the solid NMR spectrum, and the results are listed in Table 2. Most of the HA structures in the two groups of soils were O-alkyl C, and the content of O-alkyl C in the buried ancient paddy soils was relatively higher than that in the modern farming paddy soils. Alkyl C and aromatic C were second, and the relative contents of alkyl C and aromatic C in the buried ancient paddy soils were low. Carboxylic C has the lowest content, and the relative content in the buried ancient paddy soils was high. Alkyl C and O-alkyl C were the main HA structures. The aromaticity of HA in the modern farming paddy soils was greater than that in the buried ancient paddy soils, and the relative contents were 16.2–27.1 and 15.9–21.7%, respectively. The aliphaticity in the buried ancient paddy soils was greater than that in the modern farming paddy soils, and the relative contents were 78.3–84.1 and 72.9–83.8%, respectively. Moreover, the HA aliphaticity in the buried ancient paddy soils was greater than that in the modern farming paddy soils, and the structure was relatively simple (Liu et al. 2013; Liang et al. 2006).

**Table 2:** ^13^C NMR quantitative analysis of HA in paddy soil profile of Shanlonggang

Type	Soil layer	Alkyl C (0–50 ppm)	O-alkyl C (50–110 ppm)	Aromatic C (110–160 ppm)	Carboxylic C (160–230 ppm)	Aliphaticity	Aromaticity
Modern farming paddy soil	A1	23.5	39.7	23.5	13.3	72.9	27.1
	P1	22.6	43.5	20.4	13.5	76.4	23.6
	W1	21.8	51.2	14.2	13.8	83.7	16.3
	C1	21.2	50.4	13.8	14.6	83.3	16.2
Buried ancient paddy soil	A2	16.8	48.7	18.2	16.3	78.3	21.7
	P2	17.5	50.0	15.0	17.5	81.1	18.2
	W2	17.5	51.8	13.2	17.5	84.0	16.0
	C2	20.7	47.2	12.8	19.3	84.1	15.9

The alkyl C content in the modern farming paddy soils varied as follows: plow layer (23.5%) > plow pan (22.6%) > waterloggogenic horizon (21.8%) > parent material (21.2%). For the buried ancient paddy soils, the alkyl C content varied as follows: parent material (20.7%) > waterloggogenic horizon (17.5%) = plow pan (17.5%) > plow layer (16.8%). For both the modern and the ancient soil profiles, the O-alkyl C content increased with depth of the soil layers, except the parent layers, while the aromatic C content decreased with depth of the soil layers. In contrast, the carboxyl C content increased with depth of the soil layers. The aliphaticity of HA for the two soil profiles increased with depth of the soil layers, and the aromatic degree decreased with depth of the soil layers.

### ^13^C NMR spectra of the soil FA

The FA spectra in the paddy soil profile of the Shanlonggang site on the Liyang Plain (Fig. 3b) showed that the spectra of the modern farming paddy soils and buried ancient paddy soils were generally similar. Compared with the HA spectra, the FA spectra exhibited peaks near 23.3 and 29.8 ppm in the alkyl region, and the peak near 54.8 ppm in the methoxy C region was relatively weak, which may indicate that long alkyl chains are more abundant in HA. A strong peak appeared near 72.1 ppm, which corresponds to carbon atoms singly bonded to oxygen or nitrogen in alcoholic, etheric, or amine carbons in the carboxylic region (Poutanen and Morris 1985). The signal peaks of aromatic C and carboxyl C were generally consistent with the HA trends.

The results of the quantitative analysis of the relative contents of the functional groups in the chemical shift integral are listed in Table 3. The main structure in the FA of both the modern and the ancient soils was O-alkyl C, and the relative content of O-alkyl C in the buried ancient paddy soil layers was greater than that in the modern paddy soil layers. The alkyl C and aromatic C contents were second, and the alkyl C and aromatic C contents in the buried ancient paddy soils were relatively low. The lowest content was that of carboxylic C, and its content in the buried ancient paddy soils was relatively high. The relative aromaticity of FA in the modern farming paddy soils (15.8–20.9%) was greater than that in the buried ancient paddy soils (12.8–18.1%), and the relative aliphaticity in the buried ancient paddy soils (81.9–87.2%) was greater than that in the modern farming paddy soils (79.1–84.2%).

**Table 3:** ^13^C NMR quantitative analysis of FA in paddy soil profile of Shanlonggang

Type	Soil layer	Alkyl C (0–50 ppm)	O-alkyl C (50–110 ppm)	Aromatic C (110–160 ppm)	Carboxylic C (160–230 ppm)	Aliphaticity	Aromaticity
Modern farming paddy soil	A1	22.4	42.6	17.2	17.8	79.1	20.9
	P1	19.5	48.5	15.1	16.9	81.8	18.2
	W1	18.8	50.2	13.8	17.2	83.3	16.7
	C1	17.3	50.7	12.8	19.2	84.2	15.8
Buried ancient paddy soil	A2	15.7	49.3	14.4	20.6	81.9	18.1
	P2	16.0	49.5	13.7	20.8	82.7	17.3
	W2	16.5	49.5	12.5	21.5	84.1	15.9
	C2	17.1	50.9	10.0	22.0	87.2	12.8

The content of alkyl groups in the modern farming paddy soils varied as follows: plow layer (22.4%) > plow pan (19.5%) > waterloggogenic horizon (18.8%) > parent material (17.3%). The alkyl C content in the buried ancient paddy soils (15.7–17.1%) was relatively smaller than that in the modern paddy soils, which increased slightly with depth of the soil layer. The O-alkyl content increased significantly in the modern farming paddy soils with depth of the soil layer, while the aromatic content decreased with depth of the soil layer in the two groups of soil samples. The carboxyl content increased with depth of the soil layer. The aliphaticity of the two groups increased, and the aromaticity decreased with depth of the soil layer. The above results are consistent with the HA results.

### Multivariate analysis of ^13^C NMR of the soil HA and FA

**Fig. 4:**
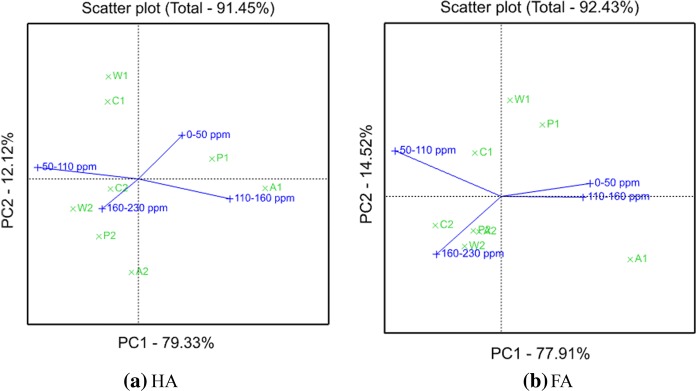
Biplot generated by principal component analysis of the NMR spectra of HAs and FAs in paddy soil profile of the Shanlonggang site

The treatment and interpretation of large amounts of data can be simplified by chemometric methods or multivariate analyses. Among these, PCA is widely applied to simplify the interpretation of chemical and spectroscopic results in complex systems (Einax et al. 1997). PCA extracted two principal components, PC1 and PC2, which accounted for 91.45% (HA) and 92.43% (FA) of the total variance. The resulting two biplots with the scores and loading plots are shown in Fig. 4.

The PC scores for soil layers A–C were differentiated, and the layers of the modern farming paddy soils (A1–C1) appeared in the first to the third quadrants of the biplot. HA (Fig. 4a) is represented in the A1–C1 layers, with a large amount of alkyl C (0–50 ppm), aromatic C (110–160 ppm), and O-alkyl C (50–110 ppm). FA (Fig. 4b) was represented in the A1–C1 layers, with a large amount of alkyl C (0–50 ppm) and O-alkyl C (50–110 ppm). Moreover, A1 was visibly separated from P1–C1 in the third quadrant due to the larger amount of carboxylic C (160–230 ppm). The buried ancient paddy soil layers A2–C2 appeared in the fourth quadrant of the biplot, showing large and negative values for PC1 and PC2, with a large amount of O-alkyl C (50–110 ppm) and carboxylic C (160–230 ppm). The biplots showed that the HA and FA spectra of the soil layers in the modern cultivated paddy soils changed significantly with the change in soil depth, especially in the A1 layer, while the buried ancient paddy soils showed little HA and FA change. This finding indicates that the layers of the modern farming paddy soils (A1–C1) were relatively rich in the aromatic region (including aromatic and phenol), and the layers of the buried ancient paddy soils (A2–C2) showed a more prominent aliphatic region (including carbohydrates, methoxyl, and O-alkyl and alkyl groups) than the modern farming paddy soils. The HA biplot (Fig. 4a) showed that the PC1 loadings were large and positive for the aromatic C (110–160 ppm) regions and large and negative for the O-alkyl C (50–110 ppm) regions. Conversely, the PC2 loadings were large and positive for alkyl C (0–50 ppm) and negative for carboxylic C (160–230 ppm). The FA biplot (Fig. 4b) showed that the PC1 loadings were large and positive for alkyl C (0–50 ppm) and negative for carboxylic C (160–230 ppm). Conversely, the PC2 loadings were positive for O-alkyl C (50–110 ppm) and negative for carboxylic C (160–230 ppm). According to the distribution of the PC loadings, HA belonged to the biplot area with the largest amount of aromatic components (large and positive PC1 loading for 110–160 ppm), and FA belonged to the biplot area with the largest amount of aliphatic components (large and positive PC1 loading for 0–50 ppm and positive PC2 loadings for 50–110 ppm). On the basis of the CPMAS ^13^C NMR spectra of HA and FA in the paddy soil profile of the Shanlonggang site, the relative abundances of the integrated regions represented the input parameters for PCA, which were sensitive to content variation among the different soil layers (Smejkalova et al. 2008). The results showed that HA contained more aromatic C than FA, the aromaticity and oxidation degrees of HA were relatively high, the structure of HA was complex, the aliphaticity of FA was stronger than that of HA, and the structure of FA was relatively simple (Cook and Landford 1998).

Overall, the results obtained by PCA were in good agreement with previous structural conclusions inferred from the subjective observation of variations in CPMAS spectra (Conte et al. 2006).

## Discussion

The total organic carbon content of the buried ancient paddy soils was similar to that of the modern cultivated paddy soils at the Shanlonggang site, indicating that the buried ancient paddy soils retained the organic carbon accumulated 3000 years ago. In ancient times, “fire” and “water” were two kinds of forces that nature endowed to mankind, and in sections of ancient rice fields, a straw-burning ash pit, residual carbonated rice, and black carbon can be found (You 1995; Ding 2004). The incineration of straw not only cleaned the field but was also convenient for planting in the following season; at the same time, most weeds were burned. The characteristics of “unified under the whole world” regarding rice in the palynological spectrum of the surface soil in the prehistoric paddy field are evidence of “water flooding” by ancestors, which eliminated most dry weeds (Lu et al. 2006; Cao et al. 2006). The residual organic matter that was originally in the burned paddy straw and in the situ paddy straw and leaves after harvesting could be converted into organic carbon with alkene groups or aromatic C to keep organic carbon in the soil for long periods of time (Greenland 1998; You 1995). Cao (2008) and Ding (2004) also found that the contents of organic matter in the surface soil of ancient paddy soils formed 6000 years ago at the archeological site were equivalent to or even slightly higher than those of local modern surface paddy soil. The ancient paddy soils had a lower organic carbon content in the plow layer and a higher organic carbon content in the plow pan, waterloggogenic horizon, and parent material layers than the modern paddy soils. Similar results were reported for the ancient paddy soils formed 3320 years ago at the Kunshan Chuodu Mountain site (Lu et al. 2006).

The difference in the organic carbon distribution between the modern and ancient paddy soils could be attributed to seasonal rainfall, alternate wetting and drying of soil, freeze–thaw action and flooding treatment, the long-term application of organic fertilizers, and other natural and human activities (Lu et al. 2006; Hu et al. 2007). The concentrations of organic carbon and total aromatic C were determined in 25 samples of bottom sediments from Lake Valencia, Venezuela. The results demonstrated that the total aromatic concentrations and the normalized total aromatic C (normalized to organic carbon) make it possible to detect organic matter of anthropogenic origin. The variation in the band intensity of the Fourier transform infrared (FTIR) spectra for some functional groups present in the saturated and aromatic hydrocarbon fractions gives an indication of anthropogenic contribution for some zones of the lake (López et al. 2000). Moreover, compared with the modern paddy soils, the buried ancient paddy soils showed a uniform organic carbon distribution. This result can be explained by the limited use of organic fertilizer in ancient times, few disturbances, and the long-term decomposition of organic matter (David and Chris 2003). These results show that the contribution of paddy wetlands to the soil carbon pool was relatively stable, and this plays an important role in the global carbon cycle and ecological environmental protection (Camille et al. 2006; Schnitzer 1978).

Compared with those in the modern paddy soils, the HA and FA contents in the buried ancient paddy soils underwent little change. However, the average HA and FA contents in the soil layers of the buried ancient paddy soils were 2.05 and 0.92, respectively, which were higher than those in the modern paddy soils (1.61 and 0.91, respectively). This finding indicates that the relative increase in HA was greater than that in FA in the buried ancient paddy soils. This discrepancy may occur because the physical and chemical properties of HA are relatively stable and there is a higher degree of aromatization. HA commonly has a higher molecular weight and higher stability than FA and is insoluble in water, and long-term burial conditions make HA more stable (Lu et al. 2006; Dou et al. 2008).

The HA/FA ratio is an important parameter for measuring the quality of soil HS, which indicates the degree of humus polymerization and humification of soils, and the greater the HA/FA ratio is, the higher the content of HA and the better the quality of the humus (Stevenson 1982). The average HA/FA values of the buried ancient paddy soil and the modern tillage paddy soil were 3.13 and 2.17, respectively. With an increase in the soil depth, the two groups of paddy soils showed an increasing trend in the HA/FA ratio, and the increasing trend in the buried ancient paddy soil profile was less evident than that in the modern paddy soil profile. The results showed that the paddy soil might have undergone a series of processes in a long-term enclosed buried environment, such as self-degradation, mineral inclusion, and mutual transformation between each component, which was beneficial for the accumulation of HA in the paddy soil (Schnitzer 1990; Seeber 2005). The HA ratio and the humification degree increased, which promoted the further condensation of FA into HA, with high aromatization and a complex structure, and increased the polymerization degree of the soil humus. The HS of the modern paddy soil were disturbed by humans, and the degree of humus polymerization was lower in the modern paddy soil than in the buried ancient paddy soil; the bioavailability was higher, which was easily exploited by microorganisms (Dou et al. 2008; Dou 2010).

SOM has a very complex structure containing both a hydrophobic backbone and different types of reactive functional groups. Ping and Lou (2019) found that the heterogeneity of SOM properties in soils from the Yangtze River Delta region affected the sorption characteristics, and this phenomenon was related to the highly aromatic moieties and the greater surface area in the soils with higher organic carbon content. The ^13^C NMR spectra of the HAs and FAs in all layers of the paddy soil profile at the Shanlonggang site were similar, indicating that the organic matter in the modern and ancient paddy soils may have similar origins and compositions (Cao 2008). However, the integral intensities of each region estimated in the ^13^C NMR spectra in the two groups of paddy soils were different. This finding indicates that the alkyl C and aromatic C contents decreased, and the O-alkyl C content increased in the modern farming paddy soils with depth of the soil layer, and the contents in the buried ancient paddy soils were relatively low, which changed slightly with depth of the soil layer. O-alkyl, alkyl, and aromatic contents can be linked to SOM stability (Hopkins and Chudek 1997); the O-alkyl C group is mainly composed of cellulose and hemicelluloses, which are not resistant to microbial decomposition. However, the O-alkyl C group is considered stable C (Baldock et al. 1997), and low O-alkyl and high alkyl and aromatic contents tend to increase SOM stability.

The decreased alkyl C in the modern farming paddy soils could have several explanations. First, due to the impact of the large extent of mechanization and the application of fertilizers in modern farming, bacteria and other microbes grazed on organic matter in the plow layer, which contained bacterial metabolites and should have made them inherently rich in alkyl C groups (Piterina et al. 2009; Hopkins and Chudek 1997; Tian et al. 2008); therefore, the microbial metabolism of carbohydrates could lead to the accumulation of alkyl C. In addition, the organic C that originated from the burning of rice straw in the field after the rice harvest was dominated by alkyl and aromatic C structures. Scientific evidence was found for “plowing with fire and weeding with flooded water,” an early paddy field management technology during the Neolithic Age in China (Cao 2008). Thus, long-term land use and management practices may increase the alkyl content in SOM and the proportion of aliphatic compounds. Overall, the transformation of O-alkyl-dominance to alkyl-dominance increases the stability of SOM (Lorenz et al. 2007).

Compared with those in the modern paddy soils, the HS in the buried ancient paddy soils had a lower aromaticity and oxidation degree and a simpler structure. The ancient paddy soils were buried in a relatively warm and humid environment with low soil activity, which was not favorable for aromatic C accumulation. Moreover, the aromaticity degree of HA and FA in the two soil profiles both decreased with increasing soil depth. This finding further supports the idea that soil formation over long time periods or under humid conditions is beneficial for aliphatic C accumulation but is not suitable for aromatic C accumulation, and the reclamation of soils would decrease aliphatic carbon and increase the accumulation of aromatic carbon in soils (Camille et al. 2006; Schnizer and Khan 1978; David and Chris 2003).

HS have been studied by scholars using different techniques and methods. HS in groundwater and aquifer sediments in Bangladesh (Bengal Delta Plain) and Taiwan (Lanyang Plain and Chianan Plain) were characterized using fluorescence spectrophotometry and FTIR spectroscopy, and the results demonstrated that the HS contain phenolic, alkane, aromatic ring, and amine groups, which tend to form metal carbon bonds with trace elements (Selim Reza et al. 2011). Bojidarka and Michael (2015) applied a quincunx systematic statistical approach for collection of soil samples, and the performance was compared with the corresponding statistical variable obtained using an independent high-performance liquid chromatography–electrospray ionization (mass spectrometry) analysis. The paddy soil at the Shanlonggang site was affected by water cultivation and multiple cropping, water–dry cultivation, fertilization, and other tillage measures in the ancient and modern soil formation processes with uncertain environmental factors. Furthermore, due to the chemical heterogeneity and spatial variability of the humus, HAs and FAs are not the only organic compounds, and studying them is very difficult. Each spectral analysis has some limitations, so these analyses can be combined with element composition, infrared spectroscopy, mass spectrometry, and other study results to form a more comprehensive and systematic understanding.

## Conclusions

The changes in the organic carbon content range in the buried ancient paddy soils were less than those in the modern cultivated paddy soils at the Shanlonggang site, and the organic carbon contents in the two soil profiles both decreased gradually with increasing soil depth. The HA content was higher than the FA content in all soil layers of the paddy soil profile, and the HA/FA ratio increased with increasing soil depth for both paddy soil profiles. Compared with the modern paddy soils, the buried ancient paddy soils had a slower humification rate and little change in their HA and FA contents in the soil profile. The results of the ^13^C NMR analysis showed that the organic carbon in the buried ancient and modern cultivated paddy soils was aliphatic. The organic carbon composition consisted of predominantly O-alkyl carbon with minor contributions from alkyl carbon, carboxyl carbon, and aromatic carbon. The aromaticity of the HA and FA in the modern and buried ancient paddy soils both decreased with increasing soil depth. These results might indicate that the HS of the buried ancient paddy soils are less aromatic and oxidized than those of the modern cultivated paddy soils, and the FA in the two paddy soils had higher aliphaticity and lower aromaticity than the HA. The results obtained by PCA were in good agreement with the structural conclusions inferred from the subjective observation of variations in the CPMAS spectra. Our results revealed that datasets from CPMAS ^13^C NMR spectra are useful for rapid, objective, and satisfactory statistical discrimination among the HS in soil samples.
